# 1465. Two- versus Three-Gram Cefazolin Dosing for Surgical Prophylaxis in Colorectal Surgery Procedures

**DOI:** 10.1093/ofid/ofad500.1302

**Published:** 2023-11-27

**Authors:** Curtis D Collins, Eric Hartsfield, Robert K Cleary, Kara K Brockhaus

**Affiliations:** Trinity Health Ann Arbor, Ypsilanti, Michigan; Michigan Medicine, Ann Arbor, Michigan; Trinity Health Ann Arbor, Ypsilanti, Michigan; Trinity Health Ann Arbor, Ypsilanti, Michigan

## Abstract

**Background:**

International guidelines recommend an elevated dose of cefazolin 3 grams for surgical prophylaxis in patients weighing ≥ 120 kg. This recommendation was made based on limited pharmacokinetic data, low cost, and favorable safety profile of cefazolin. Since the release of these recommendations nearly a decade ago few analyses have investigated the impact of these elevated dosing recommendations on surgical outcomes.

**Methods:**

A multi-center, retrospective cohort study was performed utilizing available data from a large, validated database of elective colorectal surgeries in Michigan. Adult patients weighing ≥ 120 kg who received cefazolin and metronidazole for surgical prophylaxis between July 2012 and June 2021 were included. Emergent or urgent surgeries, missing follow-up and antibiotic information, and patients who did not receive either 2 or 3-grams of cefazolin with 500 mg metronidazole were excluded. The primary objective was to evaluate the incidence of surgical site infections (SSIs) between patients receiving 2 grams versus 3 grams of cefazolin. Secondary objectives analyzed the incidence *Clostridioides difficile* infection (CDI) between cohorts. The impact of cefazolin dosing and confounders was tested through a multivariable logistic regression model.

**Results:**

Six-hundred-fifty procedures were included. Four-hundred nine patients (62.9%) received 3-grams cefazolin prophylaxis and 241 (37.1%) received 2-grams. The cohorts were similar with a significant difference in median body mass index in the 3-gram cohort (41 vs. 41; p = 0.023). There was no difference between 2- and 3-gram cohorts in SSIs (8% vs. 9%, p = 0.745) or CDI (1.2% vs. 0.7%; p =0.510). A significant difference in compliance with recommended antibiotic timing (93.8% vs. 88%; p = 0.017) was noted. Multivariable logistic regression showed 3-gram prophylaxis was not associated with a change in SSIs (adjusted odds ratio (aOR) 1; 95% CI 0.54 to 1.85; p = 0.994).

**Conclusion:**

Three-gram cefazolin dosing in combination with metronidazole in obese patients for surgical prophylaxis in elective colorectal surgery procedures was not associated with changes in SSIs or CDI.

**Disclosures:**

**Robert K. Cleary, MD, FASCRS, FACS**, Intuitive Surgical Inc: Honoraria

